# College and Hospital Notes

**Published:** 1905-11

**Authors:** 


### COLLEGE AND HOSPITAL NOTES.

Dr. ANDREW BACHUS, a graduate of Trinity College, Toronto, has come to Buffalo to take the place, as house-surgeon, of Dr. Robert Fraser, Riverside Accident Hospital, on Swan street, Buffalo. Dr. Fraser is convalescing from an attack of appendicitis, and is at the Riverside Hospital on Lafayette avenue.

---

THE German Hospital gift fair, which was held at Convention Hall, Buffalo, during the week beginning October 29, 1905, proved a conspicuous success from every viewpoint. It is understood that at least $50,000 will be realised, which will enable the hospital to make the much needed improvements, such as a wing for private patients, a nurses' college home, and other essential additions and changes.

---

AT Saint Vincent's Hospital, New York, a new wing that cost $700,000, was recently dedicated by Archbishop Farley. The building is a seven-story fireproof structure and embodies all the latest ideas in hospital equipment. In addition to accomodations for a hundred patients it contains a new operating room, said to be the finest in this city, *x*-ray and sterilising rooms and a community room for the sisters.

One floor, known as Adrian Iselin Hall, was built and equipped by the Iselin family at a cost of $35,000. The cost of erection was defrayed by the late Adrian Iselin, and two wards, the Louise Marie and St. Therese's, were furnished by Miss Louise Marie and Miss Therese Iselin. Another floor, St. Mary's Hall, was furnished by Mrs. Daniel O'Day.

The operating room was furnished by Dr. Frederick S. Dennis, and the *x*-ray room by John D. Crimmins. The sterilising room was furnished by Mrs. M. Irene O'Donohue, and Dr. Brooks H. Wells gave the machine for sterilising basins.

Other numerous and large donations were made by individuals and societies. At the dedicatory ceremonies, which were most interesting in character, Dr. Frederick S. Dennis, president of the medical board of the hospital, said that St. Vincent's was the first hospital in New York to be supported entirely by voluntary subscriptions. He stated that the hospital was opened in 1849 with thirty beds, and treated during the first year sixty-six patients. Now it has 425 beds and treated last year 22,695 patients, including those in the outpatient department.

---

THE New Saint Luke's Hospital, at Utica, the gift of Mr. and Mrs. Frederick T. Proctor, was handed over to the board of trustees, October 18, 1905, fully paid for, and completely furnished and equipped with modern medical and surgical appliances.

Dr. Willis E. Ford, Medical Director of the hospital, in writing a description of it says: "This is the largest single gift by any one family in central New York that we recall. The building is made of steel, hollow brick and tiles, and is fire-proof throughout. It ha some features that are unusual, among which are two landscape windows out of the principal word, overlooking the Mohawk valley. There is also a large solarium at the top of the house reached by an elevator. There are also two handsome operating rooms, so arranged that accident cases can be cared for without disturbing the patients who are in the house; also a smoking room, and two large parlors. These make it one of the most attractive, as well as substantial, of modern hospitals. It has a capacity of seventy-five beds, and cost about a quarter of a million.

"The ground was purchased and the entire expense was borne by the donors, the building was made under their personal supervision, and furnished in the most luxurious manner. On St. Luke's day the building was dedicated by the Bishop of Central New York and the clergy of this region, with appropriate religious services. In the evening the graduating exercises of the nurses were held, with the usual ceremonies. In making the transfer of this splendid gift the deed was turned over to the Board of Trustees of St. Luke's Hospital, who have managed the old building successfully in the past, with no conditions attached to the gift. When sufficiently endowed this institution ought to be an ideal charity."

